# Postoperative Rehabilitation Following Total Hip Arthroplasty in Achondroplasia: A Case Report Focused on Dislocation Prevention and Environmental Simulation

**DOI:** 10.7759/cureus.88271

**Published:** 2025-07-18

**Authors:** Shota Uchida, Shusuke Nojiri, Azusa Kayamoto, Hiroki Iida, Yusuke Osawa, Yasuhiko Takegami

**Affiliations:** 1 Department of Rehabilitation, Nagoya University Hospital, Nagoya, JPN; 2 Department of Orthopedic Surgery, Nagoya Central Hospital, Nagoya, JPN; 3 Department of Orthopedic Surgery, Graduate School of Medicine, Nagoya University, Nagoya, JPN

**Keywords:** achondroplasia, activities of daily living, hip dislocation prevention, physical therapy, rehabilitaition, short-limb dwarfism, short stature, skeletal dysplasia, stair climbing, total hip athroplasty

## Abstract

Individuals with achondroplasia (ACH), a skeletal dysplasia characterized by disproportionate short stature and joint laxity, often adopt greater hip flexion as a compensatory movement to navigate environments designed for individuals of average stature, such as when climbing relatively high standard steps or using standard-height furniture. This necessary adaptation, however, consequently increases the risk of dislocation following total hip arthroplasty (THA).

This case report describes the postoperative rehabilitation course of a 68-year-old Japanese woman (height: 113.7 cm, weight: 31.8 kg, body mass index: 24.6 kg/m²) with ACH and coexisting osteoarthritis who underwent a right THA via the posterior approach. The patient’s primary goal was to return to living on the second floor of her two-story house. Achieving this goal required her to learn how to negotiate stairs safely, with careful attention to minimizing dislocation risk, to ensure a safe discharge home. Early postoperative rehabilitation was specifically tailored to her short stature. It included transfer training with a footstool and gait training with a pediatric walker. A key part of the rehabilitation was early stair negotiation training, which used an adapted kneeling technique to prevent deep hip flexion. This technique simulated her home environment and was designed to reduce the risk of posterior dislocation. By postoperative day (POD) 7, the patient could walk independently with a T-cane. By POD 24, she had mastered stair negotiation, which allowed her to be discharged home.

This case highlights the critical need for highly individualized rehabilitation plans for patients with ACH undergoing THA. Successful outcomes depend on careful consideration of the patient’s specific body measurements (anthropometrics), their home environment, the early start of adapted movement training, and thorough education on how to prevent dislocation.

## Introduction

Achondroplasia (ACH) is a skeletal dysplasia characterized by disproportionate short stature, resulting from the constitutive activation of fibroblast growth factor receptor 3 (FGFR3), with an estimated global prevalence of over 250,000 individuals [1,2]. Patients with ACH are predisposed to early-onset osteoarthritis of the hip due to the dysplastic nature of their skeletal system [3]. Total hip arthroplasty (THA) has become the standard intervention for severe hip osteoarthritis that impairs activities of daily living (ADLs) and markedly reduces quality of life. However, THA in patients with ACH poses unique challenges attributable to anatomical variations and is associated with increased rates of perioperative and long-term complications [4,5].

In ACH, the small size of the hip joint constrains the selection of femoral head size, and the use of smaller heads has been associated with a heightened risk of impingement and dislocation [6-8]. Additionally, patients with ACH often exhibit joint laxity alongside short stature [9], potentially leading to joint instability and a greater risk of prosthetic dislocation [8-10]. Moreover, due to their short stature, individuals with ACH may adopt increased hip flexion angles during ADLs, which further elevates the risk of posterior dislocation, especially following THA performed through a posterior approach.

Therefore, postoperative physical therapy interventions for patients with short stature, such as those with ACH, are considered to require careful attention to the risk of dislocation due to their unique anatomical and biomechanical characteristics. In particular, the risk of posterior dislocation may be increased after THA using a posterior approach because of the increased hip flexion angle during ADLs due to short stature. In this case, early assessment of the home environment enabled training in a simulated home environment during hospitalization, and the patient was able to learn movement strategies to reduce deep hip flexion. Herein, we report a case of a patient with ACH who was successfully discharged home after THA through rehabilitation that took into account both physical characteristics and the home environment.

## Case presentation

Patient information

The patient was a 68-year-old Japanese woman with a height of 113.7 cm, a weight of 31.8 kg, and a body mass index of 24.6 kg/m². Her primary diagnosis was osteoarthritis of the hip, with comorbid conditions including ACH, lumbar spinal canal stenosis, and hypertension. The lumbar spinal canal stenosis was stable and did not significantly affect her mobility. However, she experienced neuropathic pain characterized by a tingling sensation, which was managed effectively with oral medications including prostaglandin E1, mirogabalin, loxoprofen, and tramadol. Therefore, the condition did not interfere with the postoperative rehabilitation process.

**Figure 1:**
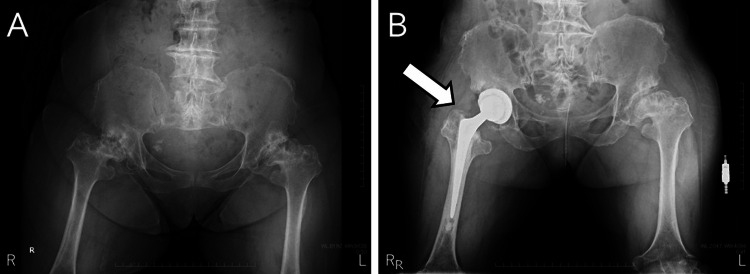
X-ray images of the hip: (A) preoperative and (B) postoperative.

During follow-up for lumbar spinal canal stenosis secondary to ACH at our institution, the patient reported right hip pain. X-ray images revealed hip osteoarthritis corresponding to grade 4 in the Kellgren-Lawrence classification. Due to the progressive worsening of right hip pain over the year preceding surgery, the patient was indicated for THA. The preoperative Japanese Orthopedic Association hip score was 28 out of 100, indicating significant impairment. The patient experienced nocturnal pain, markedly restricted range of motion, and required a cane for ambulation. ADLs, such as standing, stooping, and climbing stairs, were also severely limited. THA was performed via a posterior approach. The implants used were a Trident II 42-mm acetabular cup, X3 flat liner, Exeter 33-mm femoral stem, and a delta ceramic head with a +4-mm offset (all from Stryker, NJ), utilizing the Mako robotic-assisted system (Figure 1).

The operative time was 145 minutes, with an intraoperative blood loss of 335 mL. No intraoperative complications were observed. The patient was discharged home on postoperative day (POD) 24.

Rehabilitation course　

**Figure 2:**
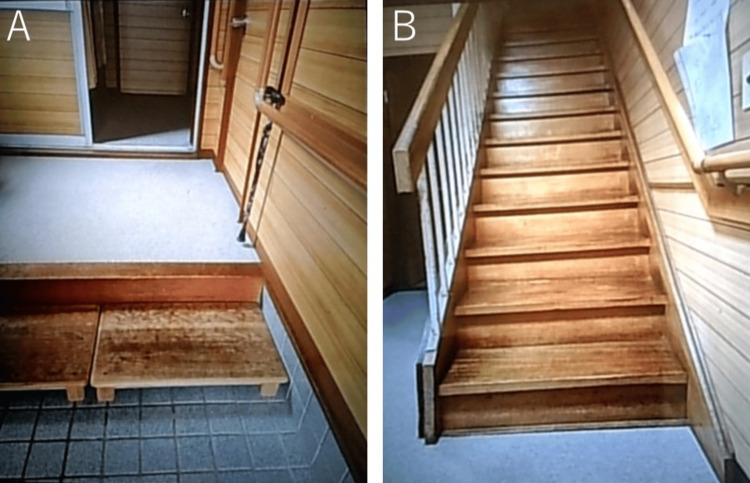
Home environment. (A) Entrance step: 22 cm high, with an additional 10-cm-high stepping stool; (B) stairs: 16 steps, each 18 cm high.

Preoperative physical function was evaluated on the day prior to surgery (Table 1). The assessment also included a detailed interview regarding the patient’s preoperative home environment and ADLs. Although the patient was independent in ADLs, she compensated for her disproportionate short stature by ascending and descending stairs on all fours and performing bed transfers in a prone position. The patient resided on the second floor of a two-story house with her parents. The entrance step measured 22 cm, and a 10-cm step stool was utilized. The staircase consisted of 16 steps, each with an 18-cm rise (Figure 2). Although relocation to the first floor was proposed, the patient preferred to continue living on the second floor due to her family's living arrangements. Hip abduction range of motion was measured in maximum hip extension. Preoperatively, hip flexion contracture was observed, and gait speed in the 10-meter walk test was reduced.

**Table 1:** Preoperative physical function assessment. MMT, manual muscle test

	Preoperative
Range of motion (degree; right/left)	
Hip flexion	100/75
Hip extension	-45/-40
Hip abduction	10/15
Hip adduction	-5/-5
Hip external rotation	20/25
Muscle strength	
Hip abduction (MMT; right/left)	3/2
Knee extension (kgf･m; right/left)	1.07/0.98
Basic mobility	
Rolling over	Independent
Living and Sitting	Independent
Rising	Independent via supine position
Walking	Utilizes T-cane in the right hand; exhibits lateral trunk flexion toward the swing leg side and pelvic elevation during the stance phase
Stair climbing	Independent

**Figure 3:**
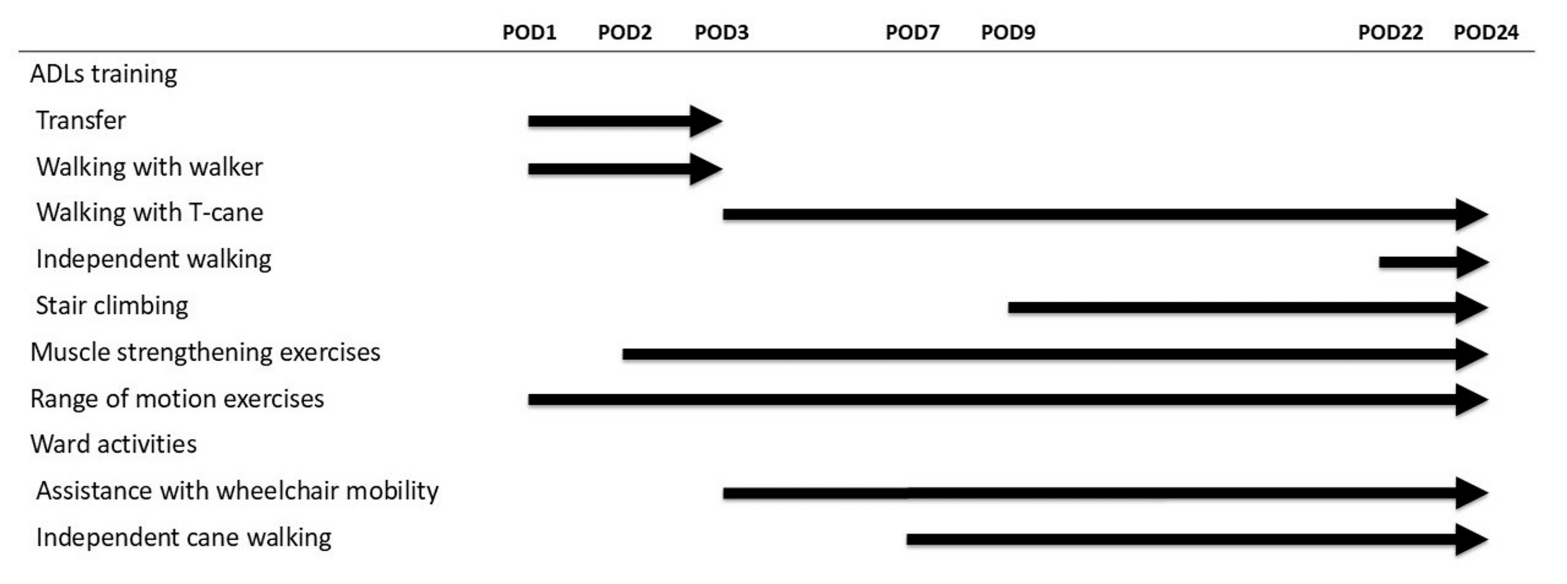
Rehabilitation progress. POD, postoperative day; ADLs, activities of daily living

**Figure 4:**
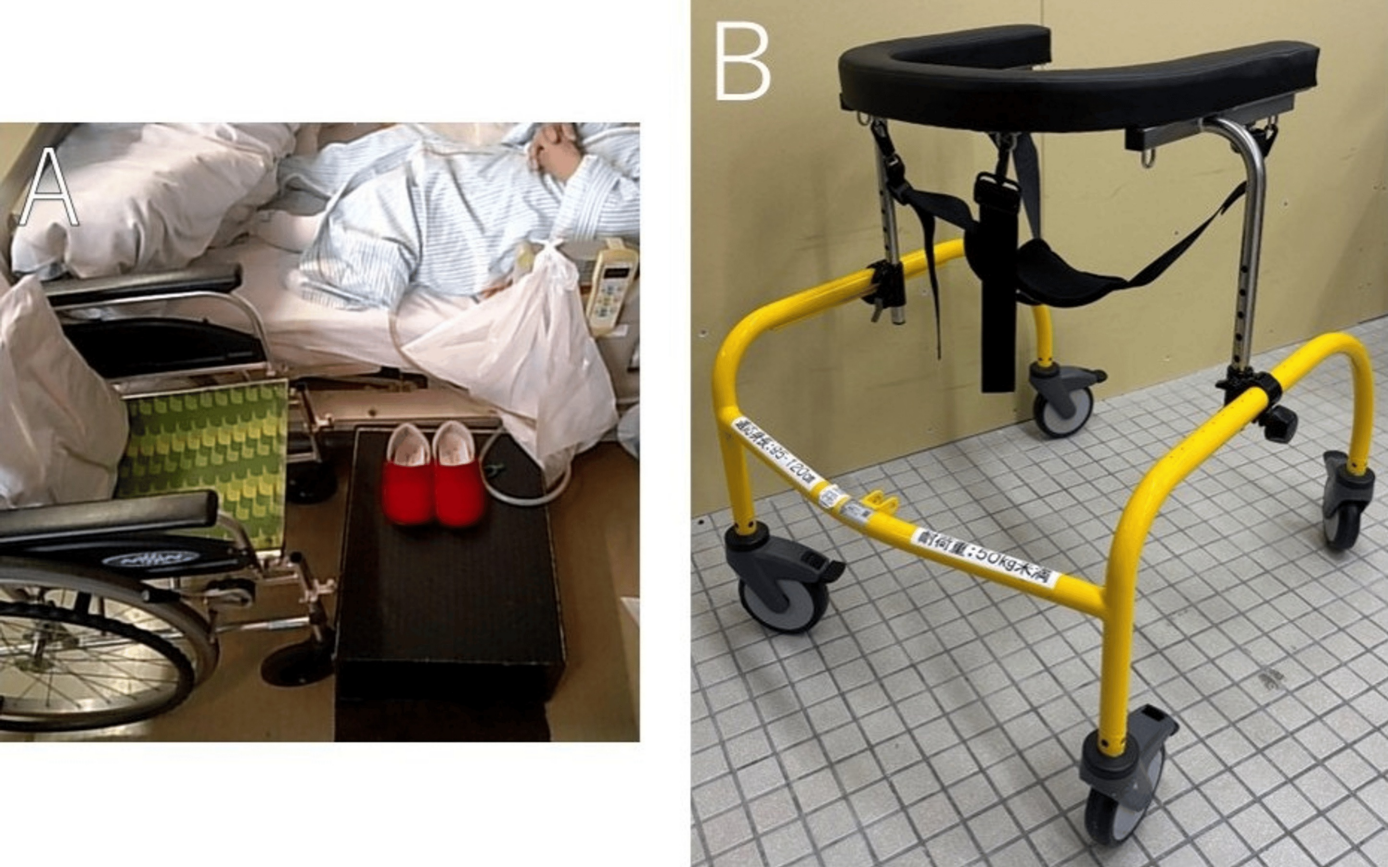
Strategies for transfer and gait training in short stature (A) Transfer using a footstool to compensate for lower limb shortening and ensure safe movement. (B) Pediatric walker adapted for short stature.

The postoperative rehabilitation course is summarized in Figure 3. Full weight-bearing was permitted from POD 1. Due to the patient's short limb length, a footstool was used to facilitate safe transfers (Figure 4A). Using a pediatric walker enabled early initiation of ambulation training (Figure 4B). Training with a T-cane began on POD 3, and independent gait for approximately 100 m was achieved by POD 7. Training for ADLs began on POD 9, focusing on stair negotiation necessary for discharge home.

Given the increased risk of deep hip flexion and posterior dislocation when ascending and descending stairs in a patient with short stature, a training environment was created to simulate the patient's home staircase. Before surgery, the patient ascended and descended stairs in a four-point crawling posture, which involved deep hip flexion. However, this method posed a high risk of postoperative posterior dislocation, and an alternative method of movement was needed. Therefore, we instructed the patient to avoid deep hip flexion by encouraging her to raise her upper body and by elevating the hand-supporting step by one level, while using the conventional crawling on all fours as the base of the movement. In the instruction, we emphasized movement strategies to avoid deep hip flexion and utilized kneeling movements for safer stair climbing (Video 1).

**Video 1:** Stair climbing exercise session.

Postoperaitive physical function was evaluated on POD 23 (Table 2). Despite the persistence of a flexion contracture, the patient's gait speed improved, and independence in basic activities, including stair climbing, was achieved, which facilitated discharge home on POD 24.

**Table 2:** Postoperative physical function assessment results. POD, postoperative day; MMT, manual muscle test

	Preoperative	POD 23
Range of motion (degree; right/left)		
Hip flexion	100/75	110/90
Hip extension	-45/-40	-20/-30
Hip abduction	10/15	30/20
Hip adduction	-5/-5	0/-5
Hip external rotation	20/15	40/15
Muscle strength		
Hip abduction (MMT; right/left)	3/2	4/3
Knee extension (kgf･m; right/left)	1.07/0.98	1.06/1.31
Basic mobility		
Walking	Independent; use of T-cane	Independent; use of T-cane
Stair climbing	Independent	Independent

## Discussion

In this case report, we described the postoperative rehabilitation process following THA in a patient with ACH and disproportionate short stature. Individualized environmental adjustments and movement instruction during early mobilization enabled a smooth transition to home discharge. This suggests that tailored physical therapy based on the patient's physical attributes and home environment is critical for successful discharge in patients with ACH.

Patients with ACH are known to have an increased risk of prosthetic hip dislocation due to joint laxity and small hip joint size [8]. Furthermore, disproportionate short stature necessitates compensatory movements involving excessive hip and knee flexion when negotiating high steps or stairs, further elevating dislocation risk. Therefore, postoperative rehabilitation requires particular caution regarding these risks.

In the present case, the patient desired to continue living on the second floor, despite concerns regarding posterior dislocation risk during stair use. Satoh et al. reported that approximately half of THA patients modify their home environments postoperatively [11]; however, in this case, no home modifications were necessary. Early assessment of the home environment, environmental simulation during therapy, and instruction in movement techniques to avoid deep hip flexion were key components of comprehensive dislocation prevention education, all of which contributed to this outcome. Thus, for patients with ACH, early assessment of the home environment, environmental simulation, and dislocation prevention training may facilitate a smooth transition to home discharge.

Regarding functional recovery, previous reports indicate a temporary decline in physical function, including stair negotiation, within one month postoperatively in patients undergoing THA for osteoarthritis [12]. However, in the present case involving a patient with ACH, gait speed improved within one month compared to preoperative levels. The early initiation of gait training and postoperative improvements in hip range of motion likely contributed to this favorable outcome. Nozaki et al. reported that gait independence is generally achieved within seven to 10 days following unilateral THA [13], and in this case, the patient was able to walk independently with a cane by POD 7. Similarly, Jeremic et al. demonstrated the feasibility of early rehabilitation in patients with ACH by initiating gait training under partial weight-bearing from POD 1 [14]. In the present case, full weight-bearing from POD 1 facilitated the early achievement of independent gait and improvements in daily functioning, highlighting the potential for early mobilization in ACH. Tailored strategies, such as the use of footstools or pediatric walkers, may support a rehabilitation trajectory comparable to that of average-height individuals undergoing THA, enabling the timely initiation of ADLs, including stair climbing.

Several limitations should be considered. First, the findings may not be generalizable to patients undergoing THA via different surgical approaches, as postoperative stability and rehabilitation outcomes could vary. Second, the patient also had osteoarthritis in the contralateral hip, which may have influenced the rehabilitation process and functional recovery. Nevertheless, in this case, posterior-approach THA combined with individualized postoperative rehabilitation led to improvements in physical function and ADLs. Physical therapy that incorporates patients’ physical characteristics and home environments plays a critical role in facilitating a smooth home discharge. To validate and expand upon our findings, further investigations involving larger sample sizes are necessary.

## Conclusions

In this report, we described the postoperative rehabilitation course following THA in a patient with ACH. Despite concerns regarding dislocation risk associated with continued stair use and living on the second floor, early assessment of the home environment, simulation training, and comprehensive dislocation prevention guidance enabled the patient to achieve safe stair negotiation and home discharge within approximately three weeks postoperatively without complications.
